# Induction and Characterization of Tetraploids from Seeds of* Bletilla striata* (Thunb.) Reichb.f.

**DOI:** 10.1155/2018/3246398

**Published:** 2018-05-13

**Authors:** Meiya Li, Bin Ding, Weipeng Huang, Jieli Pan, Zhishan Ding, Fusheng Jiang

**Affiliations:** Zhejiang Chinese Medical University, Hangzhou 310053, China

## Abstract

*Bletilla striata* (Thunb.), an ornamental and medicinal plant, is on the list of endangered plants in China. Its pseudobulb is abundant in polysaccharide and has been used for centuries as a herbal remedy. However, a recent rise in demand has placed it at risk of extinction, and therefore, research on its propagation and genetic improvement is essential. Since polyploids tend to possess advantageous qualities, we incubated* B. striata* seeds with colchicine with the aim of creating tetraploid plantlets. Aseptic seeds treated with 0.1% colchicine for 7 days showed the highest tetraploid induction rate of 40.67 ± 0.89%. Compared with the wild-type, the tetraploids could be identified by their morphological characteristics including larger stomata at a lower density, larger leaf blades, and a thicker petiole. Contents of polysaccharide and phenolic compounds were also determined in the tetraploid pseudobulbs, revealing significantly higher values than in the wild-type.* In vitro* colchicine treatment can therefore be used to successfully produce* B. striata* tetraploids with superior pseudobulbs.

## 1. Introduction

*Bletilla striata *(Thunb.) Reichb.f. (Orchidaceae) is a traditional Chinese medicinal herb widely distributed in eastern and southern Asia. In China, the pseudobulb of* B. striata* has been used for almost 2000 years to treat pulmonary edema and enhance hemostasis in the lungs and stomach. Pseudobulb powder, which can be ingested and applied externally, has also been used to treat and heal sores, burns, wounds, and chapped skin as well as to stop bleeding [1–3]. Polysaccharides have been identified as the medicinal component of the* B. striata* pseudobulb, presenting superior directed targeting, drug delivery, and controlled release [4–7]. The antitumor activity of purified polysaccharide has also been identified* in vitro* [8]. Moreover, we previously demonstrated that the phenolic compounds of* B. striata* present high 2,2-diphenyl-1-picrylhydrazyl (DPPH) radical scavenging, antioxidant, and tyrosinase inhibition activities* in vitro* [2]. However, due to rapidly rising demand in the last decade,* B. striata* is now at risk of extinction.

In the wild,* B. striata* propagates via its pseudobulb; because it lacks an endosperm, its seeds do not germinate easily. A number of studies have therefore examined seed maturation and rapid propagation in* B. striata *[9, 10]; however, few have investigated polyploidy induction. Polyploidy is an important genomic feature and widespread phenomenon that promotes evolution, variation, and plant breeding. Polyploid plants often differ from their progenitors in morphological, ecological, physiological, and cytological characteristics [11], often resulting in a superior plant with broader leaves, good quality, high yielding, and enhanced resistance to environmental stress and diseases [12, 13].

Colchicine treatment is the traditional method of polyploidy induction. Colchicine shows high affinity to tubulin, a subunit of microtubules, and inhibits spindle function during chromosome replication and cell division. Since the induction of a tetraploid of* Datura stramonium *L. [14], colchicine treatment has been widely used in* in vitro *polyploid breeding, superseding genetic modification methods. This study details a successful* in vitro* protocol for colchicine induction of* B. striata* tetraploids.

## 2. Materials and Methods

### 2.1. Plant Material

Mature seeds were obtained from fruit of* B. striata *(Thunb.) Reichb.f., which consist of small loculicidal capsules. Capsules were collected from Jiangshan city, Zhejiang province, China, and authenticated by Professor Zhensheng Yao (Zhejiang Chinese Medical University, China). The capsules were then disinfected in 75% (v/v) ethanol for 2 min followed by 15 min immersion and agitation in 0.1% (w/v) mercuric chloride. Lastly, they were washed five times in sterilized distilled water and refrigerated at 4°C until use.

### 2.2. Colchicine Treatment for Tetraploid Induction

Seeds from disinfected capsules were immersed in liquid MS medium with 30 g/l sucrose, pH 5.8, supplemented with 0, 0.05, 0.1, 0.2 or 0.4% (w/v) colchicine, respectively, and incubated for 3, 5, 7, or 9 days, respectively, in the dark at 25 ± 2°C. Ten replicates of approximately 100 seeds were used per individual incubation condition. Seeds were then washed five times with sterile distilled water and grown on MS agar medium supplemented with 30 g/l sucrose and 0.2 mg/l NAA, pH 5.8, at 25 ± 2°C with a 14 h photoperiod (135 *μ*E/m^2^s, fluorescent light). After 4 weeks growth, numbers of germinated seeds were counted and the variant aseptic buds of the seedlings were picked out. The buds were then planted on solid medium (MS + 0.2 mg/l NAA, pH 5.8) and grown for three months. Well-rooted plantlets were washed carefully, sprayed with 0.001% (w/v) bavistin, and transplanted to a greenhouse.

### 2.3. Identification of Morphological Characteristics

The ploidy level was initially determined by morphological variation, namely, slower growth, darker larger leaves, and a thicker petiole in polyploid compared with diploid seedlings. The structure of the adaxial epidermis was analyzed under a light microscope in randomly collected leaves, and the density of stomata and length and width of guard cells were analyzed at ×400 magnification in at least five microscopic fields. Root tips (2-3 mm in length) were excised from the seedlings, incubated in 2 mM 8-hydroxyquinoline at 4°C for 5 h, and then fixed with freshly prepared Carnoy's fluid (alcohol : acetic acid, 3 : 1 (v/v)) at 4°C for 24 h. The fixed root tips were then washed five times in distilled water and immersed in 1 M HCl at room temperature for about 15–20 min. Cell nuclei in the pretreated root tips were stained with phenol carbol fuchsin solution for 1 min before squashing under a cover slip and observing at ×1000 magnification. The microscopic fields were changed and representative metaphasic cell nuclei photographed.

### 2.4. Flow Cytometric Analysis of Ploidy Level

Ploidy levels of seedlings incubated with different concentrations of colchicine for different time intervals were determined using a CytoFLEX flow cytometer (BECKMAN COULTER, USA). Flow cytometric analysis was then carried out to confirm the nuclei status of the polyploids selected by morphological analysis [15, 16]. Nuclei suspensions were obtained after chopping approximately 100 mg of new growth leaf tissue using a sharp razor blade in a specific buffer on ice according to Galbraith et al. (1983) [17]. Nuclear suspensions were filtered through a 50 *μ*m nylon filter and 1 *μ*g/ml RNase A (Tiangen Biotech Co., Ltd., Beijing, China) was added to each sample. All leaves used for samples were fresh, and all processes must operate on ice, with samples kept on ice until FCM analysis.

Nuclei suspensions were centrifuged twice at 3000 rpm for 5 min. After discarding the supernatant, the pellets were then resuspended in isolation buffer with 0.1 mg/ml of Propidium Iodide (PI) (Sigma, USA) at 37°C for 15 min then analyzed using the CytoFLEX cytometer. Fluorescence emission was measured using a 488 nm long pass filter in front of a FL2 photomultiplier. Relative fluorescence intensities were acquired using a histogram of FL2 fluorescence pulse area. Approximately 20,000–30,000 chromosomes were analyzed per sample during FCM analyses of nuclei DNA content.

### 2.5. Analysis of Chlorophyll, Polysaccharide, and Total Phenolic Content

Chlorophyll from 0.3 g fresh leaves was extracted with 5 ml 80% acetone. The specific absorption of the chlorophyll was then determined at 663 and 645 nm using a photometer, and the content of chlorophyll was determined using format according to Arnon and Mackinney [18, 19]. Crude polysaccharide was extracted from 10.0 g of pseudobulb powder of tetraploids and diploids, respectively, using boiling water and deproteinized with Sevag reagent [20]. The concentration of total polysaccharide in the water phase was then determined according to the phenol-sulphuric acid method with a mannose-glucose solution (w/w, 4 : 1) standard curve [20]. Crude phenolic compounds were extracted from pseudobulb powder according to the 95% ethanol reflux method; then the total soluble phenol content was determined with the Folin-Ciocalteu reagent according to the method of Slinkard and Singleton [21, 22] with slight modifications. Briefly, samples were made up to the final volume of 2.0 ml with methanol then thoroughly mixed with 1.0 ml Folin-Ciocalteu reagent at 25°C. After 10 min, 2.0 ml of 1.0 M Na_2_CO_3_ was added, followed by mixing with intermittent shaking and incubation at 50°C for 10 min. Absorbance at 770 nm was then determined using a spectrophotometer. Each sample was measured in triplicate, and the data were expressed as gallic acid equivalent (GAE) per mg dry weight based on the standard curve of gallic acid (*R* = 0.9996).

### 2.6. Polysaccharide Characteristic and Monosaccharide Composition Analysis

The molecular weight distribution characteristics of the total polysaccharides were determined using high performance liquid chromatography and gel permeation chromatography (HPLC-GPC) on a Waters 1525 HPLC system equipped with a 2424 evaporative light scattering detector and Ultrahydrogel™120 column (Waters, USA). Analysis was carried out using 300 mM ammonium acetate solution as the mobile phase at a flow rate of 0.8 ml/min. The temperatures of the column and detector were both maintained at 35°C throughout the determination process. The crude polysaccharides prepared in process 2.5 were dissolved in the mobile phase at a concentration of 1 mg/ml, and 20 *μ*l for each sample was injected for analysis.

The monosaccharide composition of the polysaccharide was determined according to the precolumn PMP derivatization HPLC method [23]. The crude polysaccharides prepared in Section 2.5 were hydrolyzed in 1 ml of 3 M trifluoroacetic acid in 2 ml ampoule and then incubated at 130°C for 2 h. The cooled samples were centrifuged at 3000 rpm for 6 min then dried in a vacuum before dissolving the residues in 1 ml of distilled water. Next, 20 *μ*l of PMP solution (0.5 M) and 30 *μ*l of NaOH (0.3 M) were added followed by incubation at 70°C for 60 min. The samples were then cooled to room temperature, neutralized with 30 *μ*l of HCl (0.3 M), and then 1 ml of trichloromethane was added. After vigorous shaking and layering, the aqueous layer was collected and passed through a 0.22 *μ*m filter. Standard solutions of glucose and mannose were also prepared as described above. The monosaccharides were analyzed on a Dionex UltiMate™ 3000 HPLC system (DIONEX, USA) equipped with a UltiMate 3000 PAD detector and RP18 column (4.6 × 250 mm, 5 *μ*m, DIONEX). A 10 *μ*l sample eluted with an isocratic mobile phase consisting of acetonitrile and 0.1 M PBS at a ratio of 17 : 83 (pH 6.7) was injected at a flow rate of 1 ml/min and a column temperature of 35°C. Detection was then carried out at 245 nm.

### 2.7. High Performance Liquid Chromatography (HPLC) Analysis of the Main Phenolic Compounds

Powder samples (0.5 g) of tetraploids and diploids pseudobulb were precisely weighted and refluxed with 50 ml 95% ethanol for 1.5 h, respectively. The resulting filtrates were concentrated to a volume of 5.0 ml with 95% ethanol. After filtration, 10 *μ*l samples were injected into the Dionex UltiMateTM 3000 HPLC system and analyzed using PAD at 260 nm as described previously [2]. Eight purified phenolic compounds identified in the pseudobulb of* B. striata *[24, 25] were injected as standards to better understand the changes in phenolic compounds.

### 2.8. Statistical Analysis

Results were presented as mean values ± standard deviation. Paired differences were analyzed using the Student unpaired *t*-test and qualitative data between two variable groups determined by one-way ANOVA using SPSS (Version 13, SPSS Inc., Chicago, USA). Significant differences were declared at *P* < 0.05.

## 3. Results

### 3.1. Effects of Colchicine Concentration and Incubation Time on Germination

**Figure 1:**
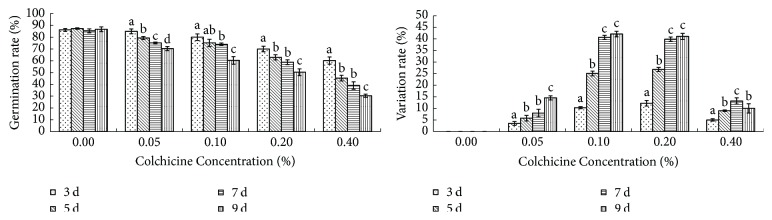
Seed germination and polyploidy induction of* B. striata* treated with colchicine for different incubation times in the dark at 25°C.* Note*. Different small letters a–d represent statistically significant difference at *P* < 0.05 comparing to each other in the same colchicine concentration treatment group.

*B. striata *(Thunb.) seeds were incubated in MS medium with different concentrations of colchicine for different incubation times and germinated on MS agar medium with 30 g/l sucrose and 0.2 mg/l NAA, at pH 5.8. The germinated seedlings were then grown on identical medium for 3 months. Variation increased and a lower germination rate was obtained with increasing colchicine and a longer incubation time, respectively. However, 0.4% colchicine treatment resulted in low germination and low variation. The variation efficiencies of 0.1 and 0.2% colchicine treatment were much higher than under all other concentrations and increased with increasing incubation time. A morphological variation frequency of 42.11% was obtained with 0.1% colchicine after incubation for 9 days (Figure 1), but a better germination rate (73.99%) was obtained with 0.1% colchicine after incubation for 7 days.

**Figure 2:**
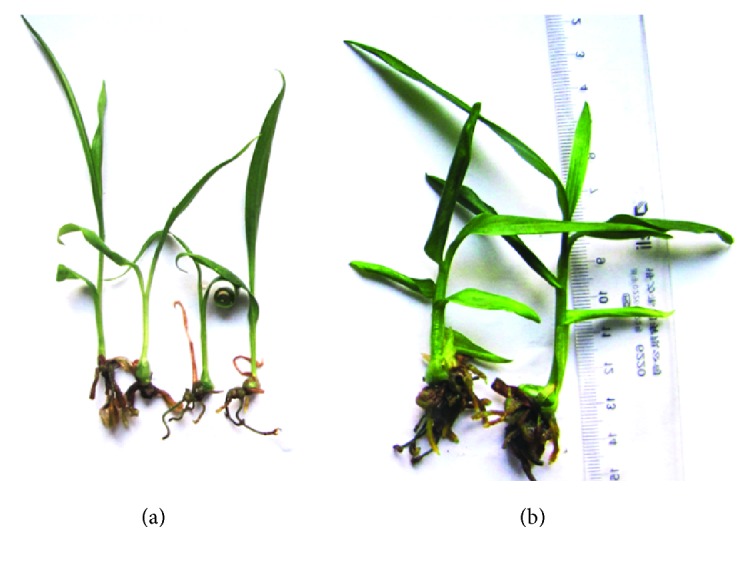
A wild-type diploid (a) and polyploid* B. striata *plantlet (b).

**Figure 3:**
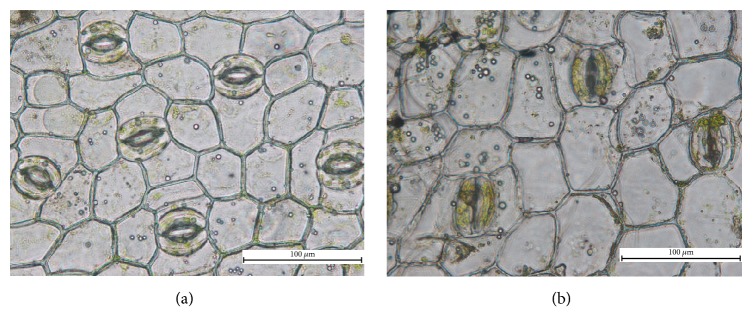
Stomata and chloroplasts from guard cells of (a) a wild-type diploid and (b) tetraploid* B. striata* plantlet (10 × 40).

Polyploidy plants presented different morphologic features, such as a thicker petiole, larger stomata, lower density of stomata across the lower epidermis of the leaves, deeper green leaf color, and thicker leaves. Figure 1 summarizes the effects of different colchicine concentrations and incubation times, and the statistical significance of the tested factors. Polyploidy plantlets were identified first by their morphological characteristics (Figure 2). Vegetative characteristics such as the length and width of the leaves were then compared with the wild-type diploid plant (Table 1). The two-sample *t*-test indicated highly significant (*P* < 0.05) differences between 3-month-old diploid (2x) and tetraploid (4x) plantlets in terms of the length and width of the leaves and the diameter of the tubers.  Figure 3 shows magnified images of the stomata. Statistical analysis revealed that the number and size of the stomata significantly differed between the diploids and tetraploids (*P* < 0.05; Table 1).

### 3.2. Tetraploid Identification

#### 3.2.1. Chromosome Counting

**Figure 4:**
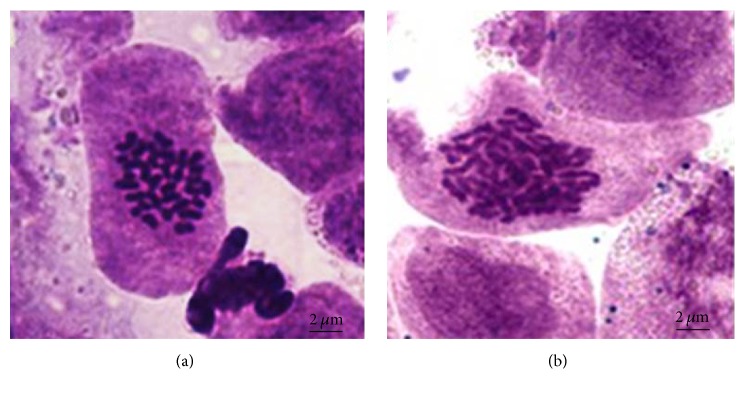
Microscopic analysis of chromosomes from root tip cells of (a) diploid (2n = 2x = 32) and (b) tetraploid (2n = 4x = 64)* B. striata *plantlet (10 × 100).

The root tips of plantlets morphologically identified as polyploids were prepared as described in Section 2.3. Phenol fuchsin stained root tips were then observed under a microscope. The chromosome number of the wide-type seedlings was 2n = 2x = 32 (Figure 4(a)) while that of the tetraploid was 2n = 4x = 64 (Figure 4(b)).

#### 3.2.2. Flow Cytometric Analysis

**Figure 5:**
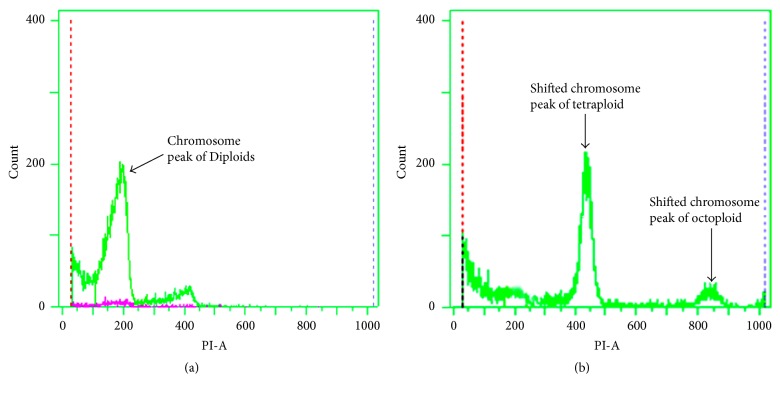
Flow cytometric analysis of nuclei from (a) diploid (2x) and (b) tetraploid (4x).* B. striata *plantlet.

The results of flow cytometric analysis are shown in Figure 4, which confirmed the results of chromosome counting. Diploid seedlings showed a large peak of diploid nuclei at 200, and a small peak of tetraploid nuclei at 400 as determined by analysis of standards with known ploidy levels (Figure 5(a)). Tetraploids, as confirmed by chromosome counting, showed a large peak shift at 400 and a small peak shift at 800 (Figure 5(b)). These results were consistent with the microscopic counts.

The tetraploid plantlets were then transplanted to a greenhouse along with the diploids and observed for three months, at which point they were found have formed robust roots and healthy leaves. Well-developed plantlets subsequently transplanted to a greenhouse for a further four months subsequently grew into healthy plants. Two years later, the chlorophyll content of the leaves and contents of phenolic compounds and total polysaccharide in the pseudobulbs were compared between the tetraploids and diploids.

### 3.3. Chemical Analysis

Chlorophyll was extracted from two-year-old leaf samples and determined by the absorbance at 663 and 645 nm according the format by Arnon [18]. The two-year-old pseudobulbs were subsequently collected, air dried, and ground into a powder, and the crude polysaccharide and total phenol content were determined. As a result, the contents of chlorophyll, crude polysaccharide, and total phenol were found to be higher in the tetraploids than in diploids (Table 2).

### 3.4. The Molecular Weight Distribution and Monosaccharide Composition of the Polysaccharides

**Figure 6:**
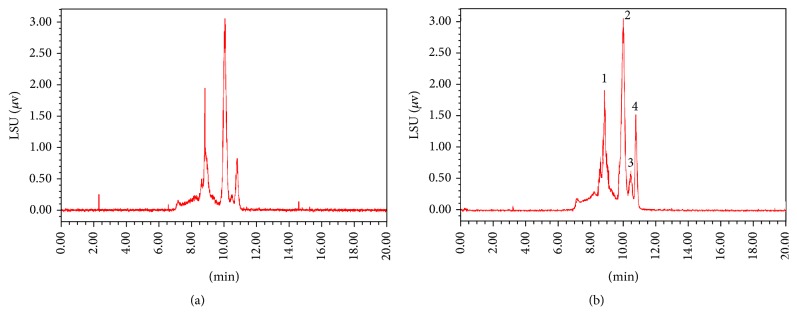
HPLC-GPC analysis of the total polysaccharide from tubers of a diploid (a) and tetraploid (b)* B. striata *plantlet.

**Figure 7:**
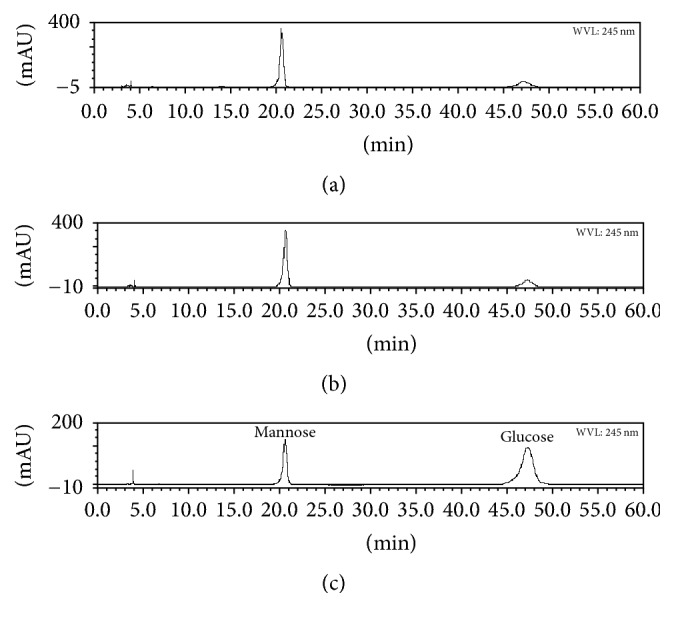
HPLC analysis of the monosaccharide composition of the polysaccharide from tubers of a diploid (a) and tetraploid (b)* B. striata *plantlet, and standards (c).

HPLC-GPC results indicated that the retention times and peak numbers of crude polysaccharides were consistent between diploid and tetraploid* B. striata *plant (Figure 6), while the areas of peak 3 and 4 were larger in the tetraploid than the diploid. Monosaccharide composition analysis (Figure 7) revealed that the crude polysaccharides of both diploid and tetraploid* B. striata* consisted of mannose and glucose, with a molar ratios of approximately 3.5 : 1, identical to a previous report [26]. These results imply that the composition and structure of polysaccharides in tetraploid* B. striata* plantlets remained unchanged.

### 3.5. HPLC Analysis of the Main Phenolic Compounds

**Figure 8:**
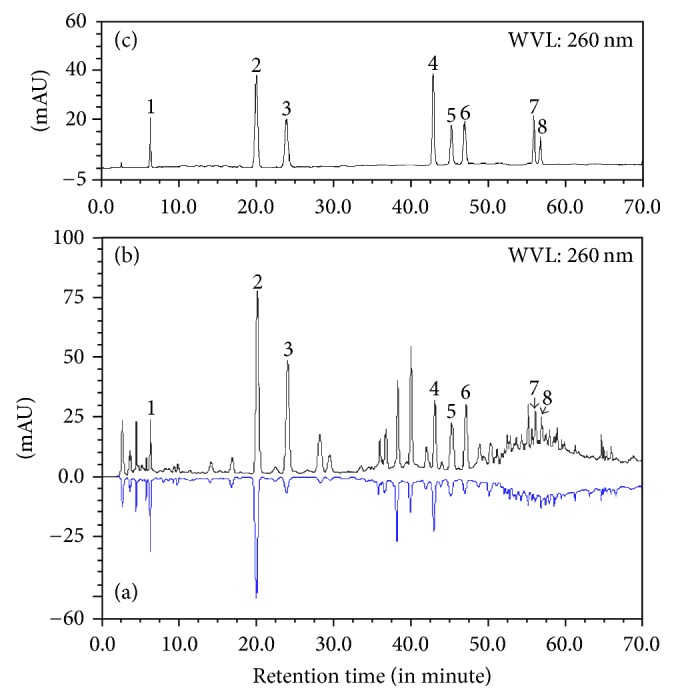
HPLC analysis of ethanol extract from tubers of a diploid ((a) in blue) and tetraploid ((b) in black)* B. striata *plantlet. (c) Standards purified from* B. striata*, peaks 1–8 represent p-hydroxybenzaldehyde, 2,7-dyhydroxyl-4-methoxy-9,10-dihydro-phenanthrene, Batatasin III, 4,4′,7,7′-tetrahydroxy-2,2′-dimethoxy-1,1′-di-phenanthrene, 4,4′,7,7′-tetrahydroxy-2,2′,8-trimethoxy-1,1′-di-phenanthrene, 4,4′,7,7′-tetrahydroxy-2,2′,8,8′-tetramethoxy-1,1′-di-phenanthrene, 3′-*O*-methyl batatasin III, and 3-hydroxy-5-methoxyl benzyl, respectively.

We previously reported HPLC analysis of crude extracts of phenolic compounds [2]. In this study, HPLC results were compared between the diploid and tetraploid plantlets (Figure 8). Most peaks seen in the diploids were also observed in the tetraploids; however, in the tetraploids, most peak areas increased significantly, consistent with the result of total phenol content.

## 4. Discussion

The percentage of flowering plants showing polyploidy is thought to be over 70% [27]. All plants sequenced to date have a repertoire of duplicated genes that have arisen via segmental duplication, transposition, tandem gene duplication, or polyploidy [28]. Moreover, polyploidy is usually beneficial to nutrient accumulation in plants.* Bletilla striata* (Thunb.) Reich b.f. has been used as a medicinal herb in China for almost 2000 years. However, in the past decade, destruction of habitats and indiscriminate excavation mean it is now under threat of extinction. In addition, insufficient cultivation has caused further depletion of the market, putting strain on the supply of* B. striata* for medicinal use. In this study, we developed a successful method for induction of polyploid* B. striata *seedlings. The polyploid plants were confirmed morphologically, via chromosome counting and through FACS analysis. They grew vigorously and developed a pseudobulb within two years. Moreover, the tetraploid leaves had a higher chlorophyll content and their pseudobulbs had a higher polysaccharide and phenolic compound content. HPLC results further revealed that the chemical components of the polyploidy* B. striata *were similar to those of the wild-type, but of a higher content. Luo et al. [29] evaluated the ploidy-dependent differences between diploid and tetraploid black locust under salinity stress by researching of physiological, ultrastructural, and proteomic traits in leaf mitochondria and found that tetraploid black locust possessed higher tolerance and stronger ability to acclimate to salinity stress than diploids. Since the polyploid* B. striata* possesses advantageous characteristics, it is possible that its metabolism-related protein expression also differs. The future aim, therefore, is to examine this using metabolomics and transcriptomics to further understand the molecular networks.

## Figures and Tables

**Table 1:** Comparison of morphological characteristics between tetraploid (4x) and diploid (2x) *B. striata *plants.

Morphological characteristics	2x	4x
Leaf length (mm)	4.51 ± 0.340	5.15 ± 0.170
Leaf width (mm)	0.43 ± 0.040	0.69 ± 0.021^*∗*^
Leaf index	10.48 ± 0.780	7.47 ± 0.410^*∗*^
Tuber diameter (mm)	3.21 ± 1.060	6.55 ± 1.430^*∗*^
Length of guard cells (*μ*m)	1.41 ± 0.408	2.11 ± 0.231^*∗*^
Width of guard cells (*μ*m)	1.08 ± 0.427	1.87 ± 0.239^*∗*^
Stomata density (per unit area)	14.00 ± 4.90	5.00 ± 0.907^*∗*^

^*∗*^represents a significant difference between mean values at *P* < 0.05 according to the two-sample *t*-test.

**Table 2:** The chemical compounds of diploid (2x) and tetraploid (4x) of *B. striata* plants.

Compounds	2x	4x
Chlorophyll (mg/g)	1.826 ± 0.509	2.409 ± 0.510
Polysaccharide (mg/g)	0.382 ± 0.024	0.455 ± 0.032^*∗*^
Total phenol content (mg/g)	4.31 ± 0.27	6.94 ± 0.38^#^

^*∗*^represents a significant difference between mean values at *P* < 0.05 according to the two-sample *t*-test. ^#^represents a significant difference between mean values at *P* < 0.001 according to the two-sample *t*-test.

## Additional Points

*Headings.* Aseptic seeds of* B. striata* were incubated with colchicine to create tetraploid plantlets with larger leaf blades, a thicker petiole, and higher contents of polysaccharide and phenolic compounds.
